# Real-World Effectiveness and Safety of Intra-Articular Polynucleotide for Knee Osteoarthritis: Large Multicenter Observational Study with Repeated Treatment

**DOI:** 10.3390/jcm15031020

**Published:** 2026-01-27

**Authors:** Wan-Ho Kim, Byung-Yoon Sung, Young-Sun Song, Jun-Seok Hong, Ho-Kwang Ryu, Kug-Jin Kim, Jong-Hoon Park, Jong-Soon Kim

**Affiliations:** 1 Department of Orthopedics, Kim Wan-Ho Orthopedic Clinic, Seoul 07702, Republic of Korea; 2 Department of Orthopedics, St. Mary’s Spine & Joint Hospital, Suwon 16271, Republic of Korea; 3 Department of Orthopedics, Hyundae Orthopaedic Clinic, Siheung 14948, Republic of Korea; 4 Department of Orthopedics, KS Hospital, Ansan 15359, Republic of Korea; 5 Department of Orthopedics, Seoul NOW Hospital, Seongnam 13591, Republic of Korea; 6 Department of Orthopedics, SMN OS Clinic, Gwangju 62058, Republic of Korea; 7 Department of Orthopedics, Whain Orthopedic Surgery Clinic, Busan 47035, Republic of Korea; 8 Department of Orthopedics, Hyosung City Hospital, Busan 48055, Republic of Korea

**Keywords:** osteoarthritis, polynucleotide, real-world evidence, repeated treatment

## Abstract

**Background/Objectives**: Intra-articular polynucleotide (PN) has emerged as an alternative to hyaluronic acid (HA) for treating knee osteoarthritis (OA), with randomized controlled trials (RCTs) reporting similar or greater pain reduction. Real-world evidence on both single- and repeated-cycle outcomes remains limited. This study evaluated PN’s real-world effectiveness and safety and whether its pain reduction falls within ranges reported in previous PN–HA RCTs, and evaluated repeated-cycle outcomes. **Methods**: Clinical data from 1048 PN-treated OA patients were retrospectively reviewed. The safety set comprised 1024 patients with follow-up visits. The efficacy set included 975 patients who completed 3–5 weekly PN injections with evaluable VAS, CGI, and PGI data at baseline, 3, and 6 months. A repeated-treatment subgroup (*n* = 45) received a second PN cycle 6 months later. First-cycle outcomes were compared with PN–HA RCTs. **Results**: In the first-cycle (*n* = 975), VAS decreased from 50.30 mm to 23.02 and 22.43 mm at 3 and 6 months (−27.28 and −27.87 mm; *p* < 0.0001), showing a comparable magnitude to RCT-reported ranges (~27–41 mm). CGI improvement was 81.0% and 79.6%, and PGI improvement 78.8% and 78.1% at 3 and 6 months. In the repeated-treatment subgroup (*n* = 45), despite a lower second-cycle baseline VAS of 31.00 mm (vs. 50.30 mm at first-cycle baseline), VAS decreased to 14.07 mm and 17.33 mm at 3 and 6 months (−16.93 and −13.67 mm; *p* < 0.001), achieving comparable absolute post-treatment pain levels. Among 1024 patients, three mild-to-moderate arthralgia events (0.29%) occurred, with no serious device-related adverse events in either cycle. **Conclusions**: PN provided meaningful 6-month pain reduction in a comparable magnitude to previous RCTs and showed consistent benefit with repeated administration without new safety concerns.

## 1. Introduction

Knee osteoarthritis (OA) is a common degenerative joint disease and a major cause of chronic knee pain and mobility limitation in aging populations worldwide [1,2,3]. Population-based studies from various countries, including Korea, similarly report a substantial burden of symptomatic and radiographic OA, with prevalence increasing markedly with age [2,3,4]. These observations highlight the need for effective intra-articular treatment options for patients who remain symptomatic despite guideline-based conservative management, including weight reduction, exercise therapy, physiotherapy, oral analgesics and non-steroidal anti-inflammatory drugs, intra-articular corticosteroid injections, and intra-articular hyaluronic acid (HA) injections [5,6,7,8,9,10,11]. However, a considerable proportion of patients experience persistent or recurrent pain despite these measures, creating ongoing demand for more durable and practical intra-articular treatment options.

In this context, polynucleotide (PN) is a highly purified long-chain DNA polymer that has emerged as an intra-articular treatment option and is thought to exert viscoelastic, extracellular matrix–supporting, and anti-inflammatory effects within the joint [12]. Several randomized controlled trials (RCTs), including head-to-head comparisons with HA, have demonstrated clinically meaningful improvements in knee pain and functional outcomes following 3–5 weekly intra-articular PN injections [13,14,15,16,17]. In these studies, pain reduction with PN was numerically greater than or similar to that with HA, suggesting its potential use as an alternative intra-articular option for patients with persistent knee OA symptoms.

Despite these encouraging findings, current RCT evidence has several important limitations. First, most PN RCTs have enrolled relatively small sample sizes. Second, they have typically evaluated only a single treatment cycle (usually 3–5 weekly injections) with follow-up restricted to approximately 2–4 months. Third, there is almost no randomized evidence on the effectiveness and safety of repeated PN treatment cycles (≥2 cycles), even though repeated intra-articular treatment is frequently undertaken in routine practice. In real-world clinical settings, patients who experience symptom relief after an initial PN cycle may undergo a second cycle when pain gradually recurs over time. Large-scale real-world evidence (RWE) is therefore essential to determine whether the benefits reported in RCTs are also observed under everyday clinical conditions, to characterize the safety of repeated exposure, and to contextualize PN’s position relative to HA injections.

In addition, patients treated for knee OA in routine clinical practice frequently present with metabolic and vascular comorbidities, such as diabetes mellitus, hypertension, dyslipidaemia, and obesity [18,19]. Although these conditions are common in OA populations, limited data are available regarding whether the safety and efficacy profile of intra-articular PN observed in controlled trials is similarly maintained in patients with such comorbidities under real-world conditions.

To address these gaps, we conducted a large multicenter retrospective observational study of patients with knee OA treated with intra-articular PN across 17 orthopedic clinics in Korea. We evaluated the effectiveness and safety of a 3–5 injection first PN treatment cycle over a 6-month follow-up period and examined whether the magnitude of pain reduction observed in routine practice falls within the ranges reported in prior randomized PN trials, including PN–HA comparative trials. In addition, we analyzed a subgroup of patients who received a second PN treatment cycle at approximately 6-month intervals to assess whether clinical benefits are maintained with repeated administration in a real-world setting. Exploratory subgroup analyses were also performed according to the presence of metabolic and vascular comorbidities to further contextualize PN outcomes in clinically relevant patient subsets.

## 2. Materials and Methods

### 2.1. Data Collection

This study was conducted as a multicenter retrospective observational review of patients treated with intra-articular PN in routine clinical practice. Clinical data were collected across 17 orthopedic clinics in Korea for all patients who received PN injections between May 2020 and October 2023. Collected variables included demographic characteristics, radiographic findings, PN injection records, follow-up assessments, and adverse event reports. All data were anonymized before analysis, and only information documented as part of routine clinical care was used.

### 2.2. Patient Selection

Patients were eligible for inclusion if they met the following criteria:

- Age ≥ 40 years;
- Diagnosis of symptomatic knee osteoarthritis based on the American College of Rheumatology (ACR) classification criteria;
- Kellgren–Lawrence (KL) grade I–III OA in the treated knee, assessed primarily on standard weight-bearing anteroposterior knee radiographs obtained in routine clinical practice;
- Receipt of 3–5 weekly intra-articular PN injections within a 6-month period.

Exclusion criteria included the following:

- No follow-up of any kind (no clinic revisit or telephone contact within 6 months).

**Figure 1:**
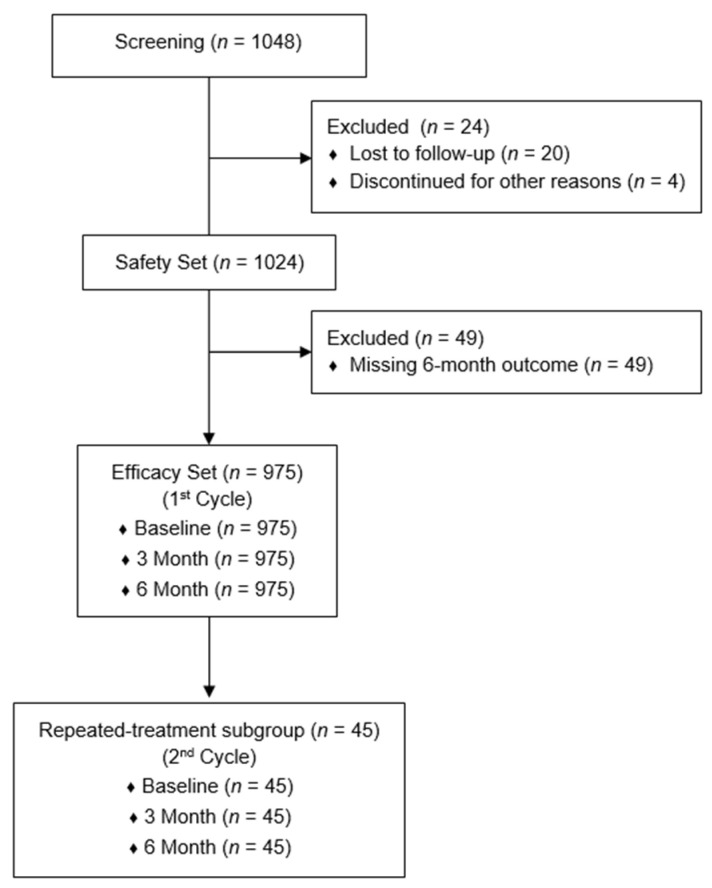
Flow of subjects throughout the study.

A total of 1048 PN-treated patients were screened, and 1024 with at least one documented follow-up were included in the Safety Set. The Efficacy Set consisted of 975 patients with complete VAS, CGI, and PGI data at baseline, 3 months, and 6 months. A subset of 45 patients received a second PN treatment cycle after the first (mean interval: 191 ± 42 days) and was analyzed separately as the repeated-treatment subgroup. The second PN treatment cycle was considered when patients experienced recurrence of knee pain during follow-up. The flow of subjects throughout the study is presented in Figure 1.

### 2.3. Ethics Approval and Informed Consent

The study was approved by the Institutional Review Board of the Korea National Institute for Bioethics Policy (IRB No. P01-202012-21-004). All procedures were conducted in accordance with the principles of the Declaration of Helsinki. Written informed consent for the clinical use of PN and for the collection and use of clinical data was obtained from all participants.

### 2.4. Outcome Measures

Clinical assessments were performed at baseline (before PN administration) and at approximately 3 and 6 months after completion of the first treatment cycle.

1. Primary endpoint
  - Safety: All adverse events (AEs) and serious adverse device events (SADEs) occurring during the study period were collected through review of clinical records and follow-up contacts.
2. Secondary endpoints
  - Pain VAS: Knee pain during weight-bearing was assessed using a 0–100 mm linear visual analog scale (0 = no pain, 100 = worst pain imaginable) [20].
  - Clinician Global Impression (CGI): Overall clinical improvement was evaluated using a 7-point scale (1 = very much improved, 2 = much improved, 3 = minimally improved, 4 = no change, 5 = minimally worse, 6 = much worse, 7 = very much worse).
  - Patient Global Impression (PGI): Patients rated their overall perception of improvement using the same 7-point scale. For analysis, CGI and PGI scores of 1–3 were classified as “improved”, and scores of 4–7 as “not improved” [21].

The same outcome measures were applied to the repeated-treatment subgroup. For this subgroup, the second-cycle baseline was defined as the assessment performed immediately before the first injection of the second PN cycle, followed by evaluations at 3 and 6 months.

**Figure 2:**
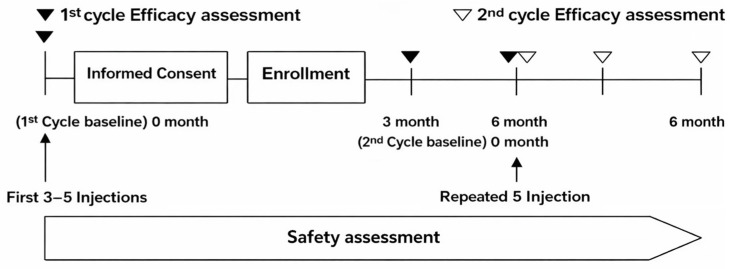
Timeline of assessments for the first and second PN treatment cycles. Baseline assessments were performed before the first cycle (3–5 weekly injections), with efficacy evaluated at 3 and 6 months. Subjects who proceeded to a second cycle after the 6-month visit were assessed on the same schedule. Safety was assessed continuously.

All participating clinics adhered to a standardized procedure for assessing VAS under weight-bearing conditions to ensure consistency across sites. An overview of the study flow is presented in Figure 2.

### 2.5. PN Preparation and Treatment Protocol

For all intra-articular injections, a single prefilled syringe containing 2 mL of a 20 mg/mL polynucleotide (PN) solution (Conjuran^®^, PharmaResearch Co., Ltd., Gangneung, Republic of Korea) was used, and the full 2 mL volume was administered to the target knee at each injection.

Because the number of injections may vary depending on patient symptoms and clinical response in routine practice, a treatment “cycle” in this study was defined as 3–5 weekly intra-articular PN injections administered within a 6-month period, reflecting real-world treatment patterns.

All injections were performed by experienced orthopedic specialists under aseptic conditions. PN was injected slowly into the joint cavity using ultrasound guidance when available or otherwise by a landmark-based approach, in accordance with routine clinical practice at each participating clinic. All syringes were single-use and discarded immediately after administration.

After each injection, patients were advised to avoid strenuous physical activity for 24 h, while routine daily walking was permitted. Oral analgesics were allowed as needed and were not otherwise restricted or monitored.

In the repeated-treatment subgroup (*n* = 45), a second PN cycle was administered using the same procedure after a mean interval of 191 ± 42 days following completion of the first cycle. The second cycle consisted of five weekly injections, with outcome assessments repeated at the second-cycle baseline and at 3 and 6 months thereafter.

### 2.6. Selection of Reference RCT Evidence

To provide context for the real-world outcomes observed in this study, previously published randomized controlled trials (RCTs) evaluating intra-articular PN were reviewed. Trials were included as external reference studies if they met all of the following criteria:

(1) Directly compared PN with hyaluronic acid (HA);
(2) Used 3–5 weekly intra-articular injections;
(3) Reported mid-term follow-up outcomes approximately 2–4 months after treatment.

These criteria align with the evidence classification described in a recent meta-analysis of PN clinical trials, which identified PN–HA comparative trials as the most methodologically reliable reference category [22]. Notably, no published PN RCT to date has reported follow-up beyond approximately 4 months; therefore, all eligible RCTs naturally fall within an early post-treatment window of ≤4 months. Three RCTs met the above criteria [16,17,23], and comparisons with the present study were accordingly focused on outcomes within this early follow-up range.

These selected RCTs served as external reference points for interpreting the effectiveness observed in the current large real-world cohort. Given differences in study design, patient populations, and follow-up timing, these comparisons should be interpreted as contextualization rather than formal comparative analysis. Because no randomized studies have evaluated repeated PN treatment cycles, between-study comparisons were limited to the first treatment cycle only.

### 2.7. Sample Size Justification

A planned enrollment target of 1200 patients was originally estimated to provide approximately 99.7% confidence for detecting adverse events occurring at a frequency of 0.5% (1 in 200 cases). In practice, 1048 patients were screened, and 1024 were included in the Safety Set, which still provided >99% confidence for detecting adverse events with a frequency of at least 0.5%. This sample size was therefore considered adequate for evaluating both safety and effectiveness in this real-world observational study.

### 2.8. Statistical Analysis

All variables were summarized using descriptive statistics (mean, standard deviation, frequency, and percentage). Changes in VAS scores from baseline to the 3-month and 6-month assessments were analyzed using paired *t*-tests for both the first and second treatment cycles. CGI and PGI outcomes were summarized as the proportions of patients classified as “improved” (scores 1–3) or “not improved” (scores 4–7). Exploratory subgroup analyses according to metabolic/vascular comorbidities were conducted using the same within-group and between-group analytical approaches.

Statistical analyses were performed using SAS version 9.4 (SAS Institute, Cary, NC, USA). A two-sided *p*-value < 0.05 was considered statistically significant, and *p*-values were reported as calculated (e.g., *p* < 0.0001, *p* < 0.001).

This study was reported in accordance with the STROBE (Strengthening the Reporting of Observational Studies in Epidemiology) statement for cohort studies, as recommended by the EQUATOR Network.

## 3. Results

### 3.1. Study Population

A total of 1048 patients were screened, of whom 1024 with at least one follow-up assessment were included in the Safety Set. Among these, 975 patients with complete VAS, CGI, and PGI data at baseline, 3 months, and 6 months comprised the Efficacy Set (first-cycle cohort).

Of the Efficacy Set, 45 patients returned within approximately 6 months after completing the first cycle and received a second PN treatment cycle; these patients were analyzed separately as the repeated-treatment subgroup.

### 3.2. Baseline Characteristics

The baseline characteristics of the Safety Set (*n* = 1024) and the repeated-treatment subgroup (*n* = 45) are summarized in Table 1. The mean age was 63.4 ± 9.0 years in the Safety Set and 64.9 ± 7.6 years in the repeated-treatment subgroup. Women were predominant in both groups. The mean BMI values were similar between groups, and the distribution of KL grades I–III reflected a real-world outpatient knee OA population seen in routine clinical practice. Patients with KL grade IV were excluded in accordance with the approved indication for intra-articular PN injection in Korea. In the first treatment cycle, the vast majority of patients in the Safety Set received five PN injections (1011 of 1024 patients), and all patients in the repeated-treatment subgroup received five injections during the second cycle. Comorbid conditions were frequently observed, particularly metabolic and vascular disorders, which are commonly encountered in routine knee OA populations. These baseline characteristics provided context for an exploratory subgroup analysis to assess whether the effectiveness and safety of PN were influenced by the presence of such comorbidities.

### 3.3. Safety Outcomes

In the Safety Set (*n* = 1024), three patients (0.29%) experienced adverse events (AEs), all of which were local injection-site pain presenting as knee arthralgia (Preferred Term) following intra-articular PN injection. Two mild events occurred within 2 days after the final injection and resolved spontaneously within 5 days; these were assessed as possibly related to PN administration. The remaining moderate event occurred approximately one month after the final injection and was assessed as unlikely to be related, with the cause recorded as unknown; the resolution date was not documented, but no sequelae were reported.

Overall, all AEs resolved without sequelae, and no serious or systemic AEs were observed. No serious adverse device events (SADEs) or unexpected PN-related AEs occurred during the study period.

In the repeated-treatment subgroup (*n* = 45), no AEs or SADEs occurred during either treatment cycle.

### 3.4. Clinical Outcomes

**Figure 3:**
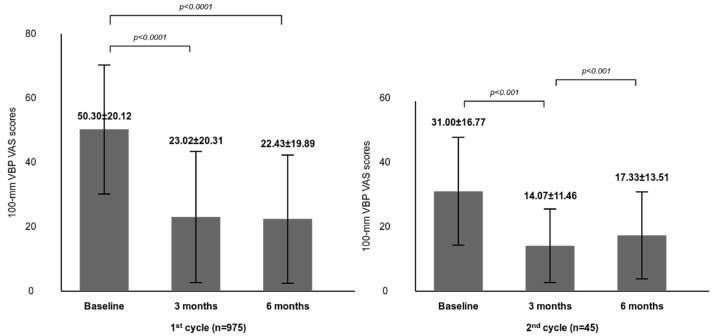
Changes in weight-bearing VAS pain scores after the 1st and 2nd PN treatment cycles. Bars represent mean ± standard deviation (SD). Comparisons were made between baseline and 3 months, and between baseline and 6 months within each treatment cycle (1st cycle: *p* < 0.0001; 2nd cycle: *p* < 0.001). The mean ± SD interval from completion of the 1st cycle to initiation of the 2nd cycle was 191 ± 42 days.

In the Efficacy Set (*n* = 975), mean VAS pain scores decreased significantly from 50.30 ± 20.12 mm at baseline to 23.02 ± 20.31 mm at 3 months and 22.43 ± 19.89 mm at 6 months (changes of −27.28 mm and −27.87 mm, respectively; both *p* < 0.0001). These reductions represent clinically meaningful improvements in knee pain (Figure 3, Table 2).

In the repeated-treatment subgroup (*n* = 45), the second-cycle baseline VAS was 31.00 ± 16.77 mm, substantially lower than the first-cycle baseline of 50.30 mm, indicating partial symptom recurrence rather than return to initial severity. VAS scores again decreased significantly to 14.07 ± 11.46 mm at 3 months (−16.93 mm) and 17.33 ± 13.51 mm at 6 months (−13.67 mm) (both *p* < 0.001). Notably, the absolute post-treatment VAS levels achieved after the second cycle were comparable to or lower than those observed after the first cycle, despite the smaller absolute reduction from a lower baseline.

At 3 and 6 months, the proportions of patients classified as “improved” (scores 1–3) were 81.0% and 79.6% for CGI and 78.8% and 78.1% for PGI, respectively. After the second cycle, improvement rates were comparable or slightly higher (CGI 84.4%; PGI 82.2–84.4%) (Table 2).

### 3.5. Comparison with Published Randomized Trials

This comparison is presented to contextualize the magnitude of pain reduction observed in the current real-world cohort relative to previously published randomized trials.

**Figure 4:**
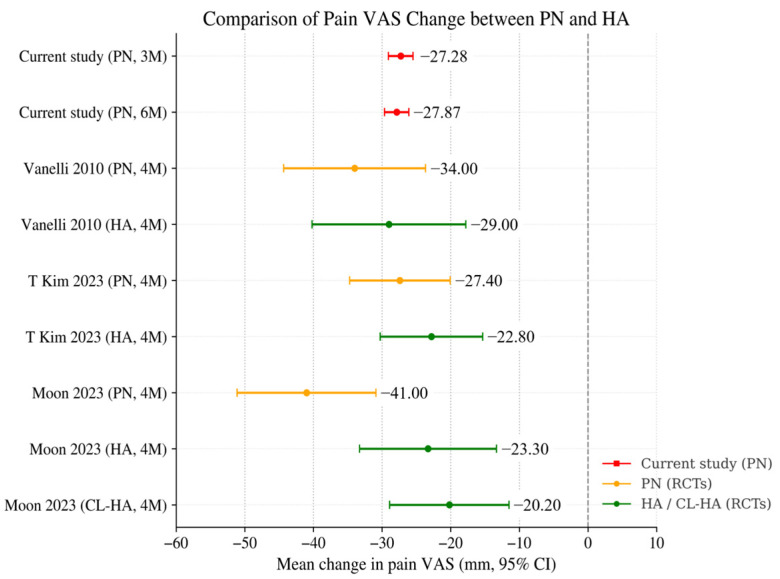
Comparison of Mean Pain VAS Change between IA PN and HA. Mean changes in weight-bearing pain VAS after intra-articular PN or HA across the current study and previously published randomized trials. Red markers represent the current study, orange represent PN arms in RCTs, and green represent HA or crosslinked HA comparators. Values are shown as mean change (mm) with 95% confidence intervals when available. The current study outcomes fall within the range of VAS reductions previously reported for PN-treated groups in RCTs, although direct comparisons should be interpreted with caution (Vanelli 2010 is [23], T Kim 2023 is [16], and Moon 2023 is [17]).

Table 3 and Figure 4 summarize key design characteristics and VAS outcomes from published randomized trials comparing PN with HA, alongside the results of the current real-world cohort. In the study by T. Kim et al. (2023), three weekly injections of PN or HA produced mean VAS reductions of −27.4 ± 17.5 mm and −22.8 ± 19.8 mm (4 months), with no statistically significant between-group difference [16]. In Moon et al. (2023), PN, non–crosslinked HA, and crosslinked HA (CL-HA) were each administered three times. PN showed greater pain reduction than both HA formulations at 4 months, with mean changes of −41.0 ± 25.8 mm (PN), −23.3 ± 27.4 mm (HA), and −20.2 ± 23.5 mm (CL-HA) [17].

In Vanelli et al. (2010), five weekly injections of PN or HA resulted in VAS reductions of −34.0 ± 28.0 mm (PN) and −29.0 ± 31.3 mm (HA) at 4 months, again with no significant between-group difference [23].

In the present real-world first-cycle PN cohort (*n* = 975), VAS reductions at 3 months (−27.28 ± 28.59 mm) and 6 months (−27.87 ± 28.31 mm) showed comparable magnitude to the range reported in PN arms of prior RCTs (approximately 27–41 mm within 4 months). Although direct numerical comparison is limited by differences in follow-up timing (2–4 months vs. 3–6 months), baseline pain severity, concomitant treatments, and differences between controlled trial settings and routine practice, the overall magnitude of improvement observed in this real-world cohort is consistent with PN effectiveness demonstrated in randomized trials.

Moreover, when examining absolute post-treatment VAS levels, rather than change scores, a consistent pattern emerges. Across all PN studies—three randomized trials and the present real-world cohort—the mean post-treatment VAS values consistently converged within a narrow range of approximately 21.0 to 23.4 mm, despite substantial variability in study design, patient populations, injection schedules, and assessment timepoints. Notably, both 3- and 6-month absolute VAS values in the present study (23.02 mm and 22.43 mm) were similar to the 4-month values reported by T. Kim et al. (23.4 mm), Moon et al. (23.2 mm), and Vanelli et al. (21.0 mm) [16,17,23]. This convergence suggests a potentially consistent pattern in residual post-treatment pain levels following PN administration, although interpretation should remain cautious given the differences between studies.

Because no randomized trials have evaluated repeated PN treatment cycles, interpretation of second-cycle outcomes relied on within-study comparison rather than external RCT benchmarks. In the repeated-treatment subgroup (*n* = 45), VAS scores decreased significantly from the second-cycle baseline, with mean reductions of −16.93 ± 20.31 mm (3 M) and −13.67 ± 21.53 mm (6 M). Importantly, despite smaller absolute reductions (reflecting the lower second-cycle baseline of 31.00 mm vs. 50.30 mm at the first-cycle baseline), the absolute post-treatment VAS levels (14.07 ± 11.46 mm at 3 M; 17.33 ± 13.51 mm at 6 M) were comparable to or lower than those achieved after the first cycle, indicating consistent pain-reducing effects without evidence of diminishing response.

### 3.6. Subgroup Analysis by Metabolic/Vascular Comorbidities

As shown in Table 4, in the first treatment cycle, patients with metabolic/vascular comorbidities had higher baseline pain scores than those without comorbidities (*p* = 0.0004). However, both groups showed significant pain reduction at 3 and 6 months, and no significant between-group differences in Mean VAS changes were observed at follow-up (3 months: *p* = 0.0727; 6 months: *p* = 0.1577).

In the repeated-treatment subgroup, pain reduction after the second cycle was again observed in both groups, with no significant between-group differences at either 3 or 6 months (*p* = 0.8670 and *p* = 0.7219, respectively). Although statistical power was limited due to the small number of patients with comorbidities in the second cycle, these findings suggest that the pain reduction effect of PN was not materially influenced by the presence of metabolic or vascular comorbidities.

Adverse events were rare and limited to mild-to-moderate local injection-site pain, with no serious or systemic events observed in either subgroup. Two events occurred in obese patients and one in a patient without documented comorbidities, and all resolved without sequelae. No comorbidity-specific safety signals were identified.

### 3.7. Additional Stratified Analyses (Gender, Baseline Pain VAS Group, and KL Grade)

Additional exploratory stratified analyses of weight-bearing pain VAS were conducted by gender, baseline pain VAS groups (0–25, 26–50, 51–75, 76–100 mm), and Kellgren–Lawrence (KL) grade I–III (Supplementary Tables S1–S3).

Both males (*n* = 243) and females (*n* = 732) showed significant within-group VAS reductions at 3 and 6 months compared with baseline (all *p* < 0.0001), with no significant between-group difference in baseline pain severity. At 6 months, mean VAS scores were significantly lower in males than in females; however, the magnitude of the difference was small and should be interpreted cautiously (Supplementary Table S1).

When stratified by baseline pain VAS group, all four groups showed significant within-group VAS reductions at both 3 and 6 months (all *p* < 0.0001). Patients with higher baseline pain levels exhibited larger absolute reductions, while post-treatment VAS levels tended to converge across groups, indicating consistent symptomatic improvement following PN treatment (Supplementary Table S2).

Across the KL grades, VAS improvements were consistently observed at both follow-up timepoints (all within-group *p* < 0.0001), with numerically larger absolute reductions in grades II–III compared with grade I; between-group differences were assessed using Bonferroni-adjusted pairwise comparisons (Supplementary Table S3).

These exploratory analyses provide descriptive insight into treatment response patterns across clinically relevant subgroups.

## 4. Discussion

This large multicenter real-world observational study demonstrates that intra-articular polynucleotide (PN) injection provides substantial and sustained reductions in knee pain over 6 months in routine clinical practice. The magnitude of pain reduction observed in the first treatment cycle (approximately −27 to −28 mm) showed a comparable magnitude to previously reported ranges from randomized controlled trials (RCTs) comparing PN with hyaluronic acid (HA), which have shown reductions of approximately 27–41 mm following 3–5 weekly injections [17,18,21]. Moreover, the absolute post-treatment VAS levels after the first cycle (approximately 22–23 mm) were also similar to those reported in PN arms of prior randomized trials, supporting a consistent pattern of PN’s pain-reducing effects across different study designs. Although these comparisons should nevertheless be interpreted cautiously, as differences in follow-up intervals, baseline symptom severity, concomitant treatments, and study environments limit the validity of direct numerical comparisons, the convergence of both relative reductions and absolute post-treatment values provides meaningful evidence that PN’s clinical benefits extend beyond controlled trial conditions into routine practice.

In addition to pain reduction, global assessments showed that approximately 79–84% of patients were classified as improved based on the Clinician Global Impression (CGI), while approximately 78–84% were classified as improved based on the Patient Global Impression (PGI) across both the first and repeated treatment cycles. These findings support the interpretation of overall symptomatic improvement within the scope of the collected outcomes in routine clinical practice. A key strength of this study is the evaluation of repeated PN administration, an aspect not investigated in prior RCTs. In the repeated-treatment subgroup, baseline pain had partially recurred by the time of the second cycle (31.00 mm vs. 50.30 mm at the first-cycle baseline), reflecting expected symptom patterns in chronic knee OA. A second PN cycle again produced significant pain reduction. The absolute reductions (−16.93 mm at 3 M, −13.67 mm at 6 M) were smaller than those observed during the first cycle, which is expected given the substantially lower second-cycle baseline. This pattern likely reflects a floor effect rather than diminished treatment response. Importantly, the post-treatment VAS levels after the second cycle (14.07 mm at 3 M, 17.33 mm at 6 M) were similar to or lower than those achieved after the first cycle (23.02 mm at 3 M, 22.43 mm at 6 M), suggesting that treatment response was maintained without attenuation upon repeated administration. These findings indicate that PN can provide consistent symptomatic benefit when re-administered in real-world clinical pathways where repeated intra-articular treatment is commonly applied.

The safety profile of PN was favorable. Among 1024 patients, only three mild-to-moderate arthralgia events (0.29%) were reported, all resolving spontaneously, and no serious adverse device events were identified in either cycle. This is consistent with the low adverse event rates observed in prior PN RCTs and supports the safety of PN use under repeated administration.

In exploratory subgroup analyses, patients with metabolic/vascular comorbidities had higher baseline pain, but VAS improvements over 3 and 6 months were broadly similar to those without comorbidities. Interpretation is limited by the small comorbidity sample in the second-cycle subgroup and the limited power for between-group comparisons.

This study has several limitations. Radiographic severity was assessed primarily using standard weight-bearing anteroposterior radiographs obtained in routine clinical practice; flexion views such as the Rosenberg view were not uniformly available across sites. Accordingly, the Kellgren–Lawrence system has known limitations in detecting joint space narrowing in the absence of osteophyte formation [24]. Other potential contributors to knee pain, such as meniscal pathology or crystal arthropathies (e.g., calcium pyrophosphate deposition disease or gout), were not systematically assessed in this retrospective real-world study. Patients with diagnosed inflammatory rheumatologic disorders were generally excluded according to routine clinical practice. Functional and quality-of-life measures (e.g., WOMAC, KOOS, EQ-5D-5L) were not collected; as such, these factors should be considered when interpreting the pain-related outcomes, while overall clinical improvement was assessed using pain VAS together with global impression measures (CGI/PGI). The repeated-treatment subgroup was small (*n* = 45) and consisted only of patients who experienced symptom recurrence and voluntarily returned for a second cycle, consistent with commonly reported retreatment patterns of intra-articular injection therapies in knee osteoarthritis [25], which limits generalizability but provides valuable preliminary evidence on repeated treatment outcomes. In addition, all participating centers were private orthopedic clinics, which may limit generalizability to other healthcare settings. The comparison with published RCTs should be interpreted cautiously, as differences in study design, patient characteristics, follow-up timing, and treatment context limit the validity of direct numerical comparisons. As with any retrospective real-world study, some degree of residual confounding is unavoidable despite standardized measurement procedures.

Nevertheless, the large multicenter cohort of more than 1000 patients and the consistent pain reduction observed with repeated PN administration strengthen confidence in the real-world effectiveness and safety of intra-articular PN. Collectively, these findings suggest that PN may represent a practical intra-articular treatment option for knee osteoarthritis, capable of providing symptomatic improvement of comparable magnitude to that demonstrated in RCTs and sustaining clinical benefit upon repeated use.

## 5. Conclusions

In this real-world, multicenter cohort of more than 1000 patients, the first cycle of intra-articular PN administered 3–5 times over a 6-month period resulted in clinically meaningful reductions in knee pain, with treatment effects comparable to the range previously reported in RCTs comparing PN with HA.

In the repeated-treatment subgroup (*n* = 45), a second PN cycle administered approximately 6 months later again improved pain and global assessment measures (VAS, CGI, PGI), with no evidence of diminished response or new safety concerns.

Taken together, intra-articular PN appears to be a practical and effective treatment option for patients with knee osteoarthritis, providing clinical pain improvement comparable to HA-based regimens and providing evidence of consistent pain improvement with repeated use in routine clinical settings. Future prospective studies—including cost-effectiveness, functional outcomes, and quality-of-life measures—are warranted to further validate and expand these real-world observations across broader clinical contexts.

## Figures and Tables

**Table 1:** Baseline characteristics of patients included in the Safety Set and the repeated-treatment subgroup. Values are presented as mean ± standard deviation (SD) or number of patients.

Variable	Safety Set (*n* = 1024)	Repeated Treatment Subgroup (*n* = 45)
Age (years old)	63.4 ± 9.0	64.9 ± 7.6
Female/Male	770/254	40/5
BMI (kg/m^2^)	24.13 ± 2.9	23.50 ± 1.8
KL grade of OA (I/II/III)	158/463/403	18/12/15
Number of injections (3/4/5)	8/5/1011	-/-/45
Comorbidities (*n* (case))	320 (469)	7 (7)
Metabolic/Vascular disorders	198 (256)	4 (4)
Diabetes mellitus	31 (31)	0 (0)
Hypertension	58 (58)	0 (0)
Dyslipidaemia	26 (26)	0 (0)
Obesity (BMI ≥ 25 kg/m^2^) *	141 (141)	4 (4)
Others (e.g., respiratory, endocrine, gastrointestinal)	165 (213)	3 (3)

*n*: number; BMI: Body Mass Index; KL: Kellgren–Lawrence grading scale. * Obesity was defined as BMI ≥ 25 kg/m^2^ in accordance with WHO Asia-Pacific classification.

**Table 2:** Changes in VAS, CGI, and PGI outcomes at 3 and 6 months following the first and second PN treatment cycles.

Variable	1st Cycle		2nd Cycle	
	3 Month (*n =* 975)	6 Month (*n* = 975)	3 Month (*n* = 45)	6 Month (*n* = 45)
Mean VAS Change (ΔVAS)	−27.28 ± 28.59 (*p* < 0.0001) **	−27.87 ± 28.31 (*p* < 0.0001) **	−16.93 ± 20.31 (*p* < 0.001) **	−13.67 ± 21.53 (*p* < 0.001) **
Mean VAS	23.02 ± 20.31	22.43 ± 19.89	14.07 ± 11.46	17.33 ± 13.51
CGI Improved (%) *	790 (81.0%)	776 (79.6%)	38 (84.4%)	38 (84.4%)
PGI Improved (%) *	768 (78.8%)	761 (78.1%)	38 (84.4%)	37 (82.2%)

* CGI and PGI are 7-point scales (1–7); improvement was defined as scores 1–3. ** *p*-values compared with each cycle’s baseline (paired *t*-test).

**Table 3:** Clinical trials comparing clinical outcomes between IA PN and HA in knee OA.

Study	Evidence Level	Patients No.	Injection Times	VAS Pain (mm)			
				Mean Changes (Months)		Mean Absolute (Months)	
Current study	observational	PN(1st) 975 PN(2nd) 45	5 (3–4) 5	−27.28 ± 28.59 (3 M) −16.93 ± 20.31 (3 M)	−27.87 ± 28.31 (6 M) −13.67 ± 21.53 (6 M)	23.02 ± 20.31 (3 M) 14.07 ± 11.46 (3 M)	22.43 ± 19.89 (6 M) 17.33 ± 13.51 (6 M)
T Kim 2023 [16]	RCT	PN 22 HA 27	3 3	−27.4 ± 17.5 (4 M) −22.8 ± 19.8 (4 M)		23.4 ± 17.6 (4 M) 28.1 ± 16.5 (4 M)	
Moon 2023 [17]	RCT	PN 25 HA 29 CL-HA 28	3 3 1	−41.0 ± 25.8 (4 M) −23.3 ± 27.4 (4 M) −20.2 ± 23.5 (4 M)		23.2 ± 24.3 (4 M) 36.0 ± 27.3 (4 M) 39.7 ± 27.3 (4 M)	
Vanelli 2010 [23]	RCT	PN 30 HA 30	5 5	−34.0 ± 28.0 (4 M) −29.0 ± 31.3 (4 M)		21.0 ± 16.0 (4 M) 21.0 ± 14.0 (4 M)	

Note: Direct head-to-head numerical comparisons should be interpreted cautiously due to differences in baseline pain severity, follow-up timing, and study design.

**Table 4:** Subgroup analysis of VAS outcomes by metabolic/vascular comorbidities.

Sub-Group	Variable	1st Cycle (*n* = 975)			2nd Cycle (*n* = 45)		
		Baseline	3 Month	6 Month	Baseline	3 Month	6 Month
With Metabolic/Vascular Comorbidities	*n*	193			4		
	Mean VAS Change	-	−29.91 ± 25.62 (*p* < 0.0001)	−30.85 ± 25.90 (*p* < 0.0001)	-	−10.00 ± 0.00 (*p* < 0.001)	−10.00 ± 0.00 (*p* < 0.001)
	Mean VAS	55.28 ± 22.07	25.37 ± 21.43	24.43 ± 22.49	25.00 ± 5.77	15.00 ± 5.77	15.00 ± 5.77
Without Metabolic/Vascular Comorbidities	*n*	782			41		
	Mean VAS Change	-	−26.62 ± 19.42 (*p* < 0.0001)	−27.13 ± 19.99 (*p* < 0.0001)	-	−15.64 ± 16.03 (*p* < 0.001)	−14.02 ± 14.15 (*p* < 0.001)
	Mean VAS	49.07 ± 19.42	22.44 ± 19.99	21.94 ± 19.18	31.59 ± 17.41	13.97 ± 11.93	17.56 ± 14.06
Between-group *p*-value		0.0004 **	0.0727 *	0.1577 **	0.4599 *	0.8670 *	0.7219 *
Power Analysis (1 − β)		-	0.5028	0.5840	-	0.1044	0.0853

*p*-values for within-group changes were calculated using paired *t*-tests comparing each follow-up visit with baseline. Between-group comparisons of change scores were performed using * pooled or ** Satterthwaite methods, as appropriate. Power analysis was conducted on the magnitude of change comparing patients with metabolic/vascular comorbidities and those without such comorbidities.

## Data Availability

The data that support the findings of this study are available on request from the corresponding author. The data is not publicly available due to privacy or ethical restrictions.

## Supplementary Materials

The following supporting information can be downloaded at https://www.mdpi.com/article/10.3390/jcm15031020/s1: Supplementary Table S1. Weight-bearing Pain VAS by Gender. Supplementary Table S2. Weight-bearing Pain VAS by Baseline Pain VAS group. Supplementary Table S3. Weight-bearing Pain VAS by KL Grade.

## Abbreviations

The following abbreviations are used in this manuscript:

ACR	American College of Rheumatology
AE	Adverse event
BMI	Body mass index
CGI	Clinician Global Impression
CL-HA	Crosslinked hyaluronic acid
EQ-5D-5L	EuroQol 5-Dimension 5-Level
HA	Hyaluronic acid
IA	Intra-articular
IRB	Institutional Review Board
KL	Kellgren–Lawrence
KOOS	Knee injury and Osteoarthritis Outcome Score
OA	Osteoarthritis
PGI	Patient Global Impression
PN	Polynucleotide
RCT	Randomized controlled trial
RWE	Real-world evidence
SADE	Serious adverse device event
SD	Standard deviation
VAS	Visual analog scale
WOMAC	Western Ontario and McMaster Universities Osteoarthritis Index
3 M	3 months
6 M	6 months
