# Supplementary material for: Real-World Effectiveness and Safety of Intra-Articular Polynucleotide for Knee Osteoarthritis: Large Multicenter Observational Study with Repeated Treatment
Source: J Clin Med. 2026 Jan 27;15(3):1020. doi: 10.3390/jcm15031020 (PMC12898195; doi:10.3390/jcm15031020)
Supplement: Supplementary file 1 [file jcm-15-01020-s001.zip › jcm-4053584-supplementary.pdf]

Supplementary Table S1. Weight-bearing Pain VAS by Gender

Gender	Variable	Baseline	1st cycle(n=975) 3 month	6 month
Male	n		243	
	Mean VAS Change	-	−29.74 ± 20.94 (P<0.0001)*	−30.72 ± 21.27 (P<0.0001)*
	Mean VAS	50.70 ± 19.50	20.96 ± 18.74	19.98 ± 19.09
Female	n		732	
	Mean VAS Change	-	−26.46 ± 20.73 (P<0.0001)*	−26.92 ± 21.28 (P<0.0001)*
	Mean VAS	50.16 ± 20.33	23.71 ± 20.77	23.25 ± 20.09
Between-group P-value**		0.714	0.055	0.023

* Within-group comparisons versus baseline were conducted using paired t-tests.
**Between-group comparisons were performed using independent-samples t-tests with Satterthwaite correction.

Supplementary Table S2. Weight-bearing Pain VAS by Baseline Pain VAS group

Baseline Pain Group (mm)	Variable	Baseline	1st cycle(n=975) 3 month	6 month
0–25	n		94	
	Mean VAS Change	-	−5.05 ± 11.83 (P<0.0001)* [2],[3],[4]**	−3.51 ± 12.42 (P<0.0001)* [2],[3],[4]**
	Mean VAS	18.11 ± 5.06	12.33 ± 10.45	10.54 ± 11.23
26-50	n		484	
	Mean VAS Change	-	−24.31 ± 15.24 (P<0.0001)* [1],[3],[4]**	−24.28 ± 14.48 (P<0.0001)* [1],[3],[4]**
	Mean VAS	39.42 ± 6.84	18.25 ± 15.32	17.51 ± 14.88
51-75	n		251	
	Mean VAS Change	-	−31.69 ± 20.80 (P<0.0001)* [1],[2],[4]**	−34.65 ± 21.91 (P<0.0001)* [1],[2],[4]**
	Mean VAS	62.15 ± 7.21	27.54 ± 20.11	26.82 ± 19.54
76-100	n		146	
	Mean VAS Change	-	−43.84 ± 25.36 (P<0.0001)* [1],[2],[3]**	−43.75 ± 25.89 (P<0.0001)* [1],[2],[3]**
	Mean VAS	83.23 ± 4.88	32.41 ± 21.05	31.51 ± 20.33

* Within-group comparisons versus baseline were conducted using paired t-tests.
** Inter-group comparisons of Mean VAS Change were performed using Bonferroni correction
[1] p < 0.0083 vs Group 1 (0–25)
[2] p < 0.0083 vs Group 2 (26–50)
[3] p < 0.0083 vs Group 3 (51–75)
[4] p < 0.0083 vs Group 4 (76–100)

Supplementary Table S3. Weight-bearing Pain VAS by KL Grade

KL Grade	Variable	Baseline	1st cycle(n=975)<br>3 month	6 month
Grade Ⅰ	n		150	
	Mean VAS Change	-	- 21.63 ± 20.95<br>(P<0.0001)*<br>[3]**	- 20.77 ± 22.35<br>(P<0.0001)*<br>[3]**
	Mean VAS	39.23 ± 21.70	17.60 ± 12.83	18.47 ± 13.35
Grade II	n		441	
	Mean VAS Change	-	- 29.15 ± 21.81<br>(P<0.0001)*<br>[3]**	- 29.45 ± 21.24<br>(P<0.0001)*<br>[3]**
	Mean VAS	50.33 ± 19.76	21.18 ± 19.74	20.88 ± 19.42
Grade III	n		384	
	Mean VAS Change	-	- 27.32 ± 19.20<br>(P<0.0001)*<br>[1], [2]**	- 28.82 ± 20.52<br>(P<0.0001)*<br>[1], [2]**
	Mean VAS	54.58 ± 18.19	27.26 ± 22.44	25.77 ± 21.98

* Within-group comparisons versus baseline were conducted using paired t-tests.
** Inter-group comparisons of Mean VAS Change were performed using Bonferroni correction
[1] p < 0.017 vs KL-grade I
[2] p < 0.017 vs KL-grade II
[3] p < 0.017 vs KL-grade III
